# Successful Therapy of Ventricular Rupture by Percutaneous Intrapericardial Instillation of Fibrin Glue: A Case Report

**DOI:** 10.1155/2013/412341

**Published:** 2013-06-27

**Authors:** Florian Willecke, Christoph Bode, Andreas Zirlik

**Affiliations:** University Heart Center Freiburg, Cardiology and Angiology I, 79106 Freiburg, Germany

## Abstract

Rupture of the ventricular myocardium is an often lethal complication after myocardial infarction. Due to the dramatic hemodynamics and the short time frame between ventricular rupture and surgical closure of the defect, additional therapeutic strategies are needed. Here we report the successful therapy of ventricular rupture by percutaneous intrapericardial instillation of fibrin glue in a 72-year-old male patient with postinfarct angina secondary to anterior myocardial infarction.

## 1. Case Report

Rupture of the ventricular myocardium after myocardial infarction is a dramatic and often lethal complication. Due to the dramatic hemodynamic dysfunction, immediate therapies are imperative. As surgical repair of the defect is often not available, percutaneous intrapericardial instillation of fibrin glue can be an alternative.

**Figure 1:**
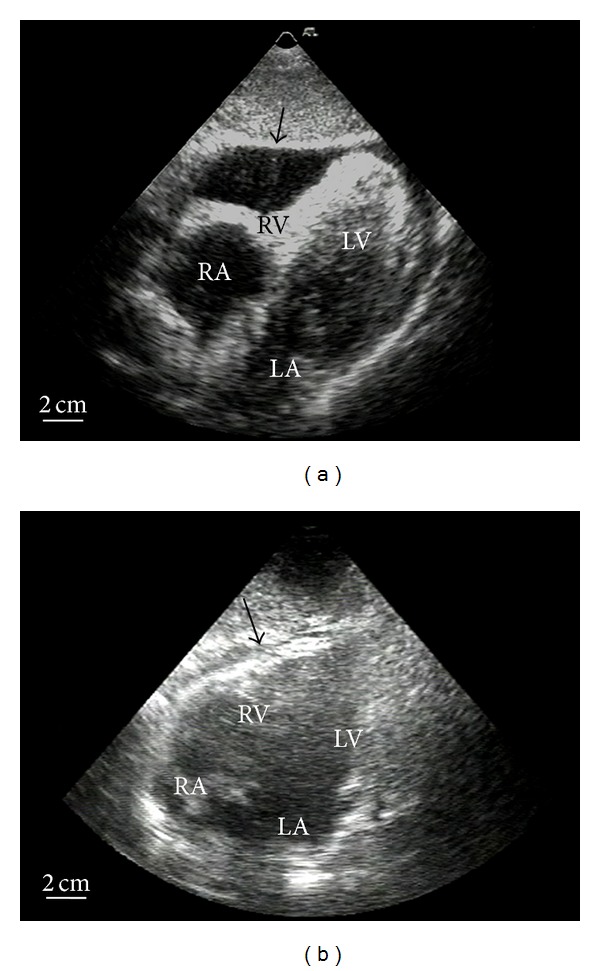
(a) Before treatment: pericardial tamponade with hemodynamic relevance on right ventricle (see arrow). (b) After successful treatment: a stable minor pericardial effusion without hemodynamic relevance (see arrow). RA: right atrium; RV: right ventricle; LA: left atrium; LV: left ventricle.

**Figure 2:**
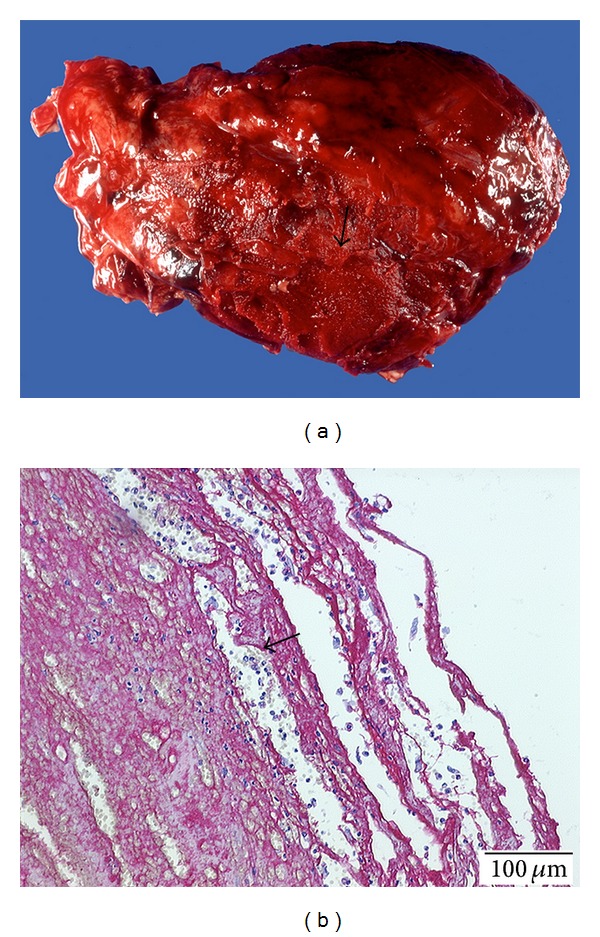
(a) Anterior view of the heart *ex vivo*: areas covered with fibrin glue resulting in a partial fibrinous pericarditis of the anterior wall (see arrow). (b) Factor VIII staining of fibrin clot: fibrin clot shows disseminated infiltration by lymphocytes and macrophages as commonly found in fibrinous pericarditis.

A 72-year-old male patient with postinfarct angina secondary to anterior myocardial infarction was transferred to our center from a community hospital after administration of systemic thrombolytic therapy using streptokinase. Coronary angiography showed single vessel disease with high grade stenosis of the LAD. Stent implantation was successfully performed with uncomplicated postinterventional course. On day three, the patient developed another episode of angina. Recatheterization excluded acute restenosis or stent thrombosis. On the same day, the patient developed rapid onset cardiogenic shock with need for resuscitation, intubation, high dose catecholamine treatment, and an intra-aortic balloon pump. Echocardiography showed an acute pericardial tamponade suggesting a ventricular rupture (Figure 1(a)). Pericardiocentesis was performed, and large amounts of blood could be aspirated and were directly retransfused. Hemodynamics stabilised only under constant aspiration. As *ultima ratio*, we instillated a total of 30 mL of a two-component fibrin glue normally used for bleeding ulcers in gastroenterology. This resulted in a sustained hemodynamic stabilization. The patient could be weaned off the balloon pump and catecholamines in the following three days. Echocardiography showed a stable minor pericardial effusion of 100 mL without any signs of hemodynamic relevance (Figure 1(b)). Unfortunately, on day nine, the patient gradually developed signs of progressive cardiogenic shock again with the need of cathecolamine treatment and finally died from pump failure on day 13. Serial echocardiographic evaluations were negative for relevant pericardial effusion. Autopsy revealed a fibrin glue induced focal peri-epicardial adhesion and extensive anterior myocardial infarction with rupture near the apex covered by the fibrin glue (Figures 2(a) and 2(b)). No relevant pericardial effusion was found.

## 2. Discussion

Rupture of the ventricular wall is an often lethal complication of acute myocardial infarction. Besides myocardial infarction ventricular rupture has also been reported after cardiac trauma, valve replacement, and cardiac tumors. In the setting of myocardial infarction ventricular rupture occurs in 2%–6% of all patients and is third to cardiogenic shock and arrhythmias as the leading cause of death after myocardial infarction [1]. The majority (90%) of ventricular wall rupture occurs within nine days of infarction: 27% within the first day and 26% after 6 to 9 days. Although thrombolytic therapy contributes significantly to reperfusion and overall survival, it slightly increases the risk for early ventricular rupture [2]. First described by William Harvey in 1647, successful surgery of left ventricular rupture after myocardial infarction was first reported by Fitzgibbon et al. in 1972 [3]. Surgical techniques include resection of the necrotic area and closure of the perforation with Teflon either by suture or glue [1]. Over the last 30 years, the incidence of cardiac rupture has declined mostly likely due to increasing use of reperfusion strategies and adjunct medical therapy [4]. Despite some advances in the surgery of ventricular rupture, mortality remains high with reported mortality rates ranging between 75% and 90% [4]. This high mortality rate is likely due to the short time frame between ventricular rupture and surgical closure of the defect. Acute cardiac surgery is not always available, and most patients have severe left ventricular dysfunction further contributing to the already severely limited prognosis. Although Figueras et al. reported that long-term survival of selected patients with prompt hemodynamic recovery after left ventricular wall rupture is possible without surgical repair [5], the devastating hemodynamics of the majority of patients warrants immediate treatment. 

When immediate heart surgery is not available or applicable as in our case, intrapericardial injection of fibrin glue as reported here facilitates an alternative treatment in this desperate situation. Autopsy revealed focal peri- and epicardial adhesion of the defect by fibrin glue, proving its effectiveness. Murata et al. reported similar results on autopsy after intrapericardial fibrin injection [6]. Intrapericardial fibrin injection was first reported in 1995 by Ogiwara et al. [7] after the same group had tested the feasibility in dogs. While there have been sporadically reports of interpericardial fibrin injection over the last 20 years [6, 8], there are only two small descriptive case report studies reporting the follow-up of patients with left ventricular wall rupture that received intrapericardial fibrin glue injections [9, 10]. Survival rates were 31% and 55% with a total of 28 patients studied. However, a considerable number of patients died before fibrin glue could be administered. 

## 3. Conclusion

This and the few other case reports on the medical treatment of left ventricular wall rupture are by far not sufficient to provide a general recommendation; however, due to its dramatic presentation and often lethal outcome, intrapericardial fibrin injection should be considered in patients with an oozing rupture of the ventricular wall as a last resort.
